# Daytime symptoms of chronic obstructive pulmonary disease: a systematic review

**DOI:** 10.1038/s41533-020-0163-5

**Published:** 2020-02-21

**Authors:** Ioanna Tsiligianni, Janwillem W. H. Kocks

**Affiliations:** 1 Department of Social Medicine, School of Medicine, University of Crete, Heraklion, Crete Greece; 2 Groningen Research Institute for Asthma and COPD (GRIAC), University Medical Center Groningen, University of Groningen, Groningen, The Netherlands; 3 Observational and Pragmatic Research Institute, Singapore, Singapore; 4 General Practitioners Research Institute, Groningen, The Netherlands

**Keywords:** Chronic obstructive pulmonary disease, Quality of life, Respiratory signs and symptoms

## Abstract

There is no single source of compiled data on symptoms experienced by patients with chronic obstructive pulmonary disease (COPD) when awake and active throughout the day. The aim of this systematic review was to evaluate the prevalence, variability, and burden (i.e., bothersomeness and/or intensity), and the impact of daytime COPD symptoms on other outcomes. The review also evaluated the impact of interventions and the measures/tools used to assess daytime COPD symptoms in patients. A systematic literature search was conducted using the primary search terms “COPD”, “symptoms”, and “daytime” in EMBASE®, MEDLINE®, MEDLINE® In-Process, and CENTRAL in 2016, followed by an additional search in 2018 to capture any new literature that was published since the last search. Fifty-six articles were included in the review. The accumulated evidence indicated that the symptomatic burden of COPD appears greatest in the morning, particularly upon waking, and that these morning symptoms have a substantial impact on patients’ ability to function normally through the day; they also worsen quality of life. A wide variety of tools were used to evaluate symptoms across the studies. The literature also confirmed the importance of pharmacotherapy in the management of daytime COPD symptoms, and in helping normalize daily functioning. More research is needed to better understand how COPD symptoms impact daily functioning and to evaluate COPD symptoms at well-defined periods throughout the day, using validated and uniform measures/tools. This will help clinicians to better define patients’ needs and take appropriate action.

## Introduction

Chronic obstructive pulmonary disease (COPD) is a common disorder characterized by persistent respiratory symptoms and airflow limitation, and is associated with significant morbidity and mortality.^1^ In 2012, more than 3 million people died of COPD, accounting for 6% of all deaths globally, and projections suggest that COPD will be the third leading cause of death in the world by 2020.^1^ The primary symptoms of COPD are breathlessness, cough, and increased sputum; many patients also experience wheezing and chest tightness or congestion, the latter particularly at times of exertion.^1^ Other secondary symptoms and related comorbidities include sleep disorders/disturbances and associated daytime sleepiness,^2,3^ increased anxiety and/or depression,^4,5^ and in severe and very severe cases, fatigue, weight loss, and anorexia.^6^

Over the past 30 years, studies have recognized that the presence of variations in circadian and diurnal lung function generally coincide with exacerbations in patients with COPD.^7–11^ The prevalence and/or burden of COPD symptoms have been shown to reflect this circadian and diurnal variability in their tendency to fluctuate over time throughout the day and night.^4,12–14^ Moreover, temporal symptom spikes, especially those occurring in the early morning and late at night, have been associated with some of the aforementioned secondary symptoms or comorbidities (e.g., sleep disturbance and increased anxiety or depression^4^). These spikes have also been linked with worse overall health status^4,15,16^ and reduced ability to complete normal daily activities.^15,17^

The published literature on COPD is extensive; however, there is substantial heterogeneity in the results reporting on the circadian and diurnal variability of COPD symptoms. A nonsystematic review article evaluating variability of COPD symptoms found that symptoms are variable over time, and can change with the seasons, within a week, and even over the same day.^18^ Moreover, comprehensive reviews looking at symptoms of COPD at specific time points during the day or night have provided useful insight that morning is the worst time of day for patients;^19–21^ however, there remains no single source of compiled data on symptoms experienced throughout the day, when the patient is awake and active. On this basis, we conducted a systematic literature review to inform on the current evidence regarding the burden of daytime symptoms of COPD. Primary objectives were to evaluate the following: (1) the prevalence, variability, and relative burden (i.e., bothersomeness and/or intensity) of daytime COPD symptoms; (2) the impact of daytime COPD symptoms on other outcomes (e.g., daily-life activities, health status, quality of life (QoL), and exacerbations); (3) the impact of interventions used to treat daytime symptoms, including pharmacotherapy; and (4) the measures and tools used to assess daytime COPD symptoms.

## Methods

A systematic literature review was conducted in accordance with the principles recommended in the Preferred Reporting Items for Systematic Reviews and Meta-Analyses (PRISMA) statement.^22^ The prespecified protocol can be found in the Supplementary File.

### Literature searches

Four electronic biomedical literature databases (EMBASE®, MEDLINE®, MEDLINE® In-Process, and CENTRAL) were reviewed in September 2016. In order to keep the findings up to date, an additional search was performed in June 2018 to capture any new literature that had been published since September 2016. Primary search terms were “COPD”, “symptoms”, and “daytime”; these were also expanded to include all recognized variations and subheadings. Searches were limited to articles for which full-text publications were available in English. Articles in other languages were excluded. Electronic searches were supplemented by manual searching of the reference lists and bibliographies of relevant systematic reviews and study publications. The detailed search strategies are provided in Supplementary Tables 1–3.

### Study selection

Titles and abstracts were initially examined for possible inclusion by two independent reviewers. A second eligibility screening—of the articles that were full-text—was then conducted by a further two independent reviewers. Any discrepancies between the decisions of the two reviewers at both stages were resolved by a third independent reviewer. The detailed selection criteria were in line with Patient, Intervention, Comparator, Outcomes, and Study Design (PICOS) and PRISMA guidelines, and are provided in Table 1.

**Table 1:** Study selection criteria.

	Criteria^a^
Patient	• Adult patients (>40 years) of any gender or race, with a diagnosis of COPD (any disease severity) and having daytime COPD symptoms • Studies enrolling a mixed population of patients experiencing daytime and night-time symptoms were only included if subgroup data for patients with daytime symptoms were available
Intervention	• No selection criteria
Comparator	• No selection criteria
Outcome	• Primary outcomes of interest included: prevalence of daytime COPD symptoms; relative bothersomeness/troublesomeness or intensity of symptoms; how the prevalence and bothersomeness/troublesomeness or intensity fluctuates throughout the day; relationship between daytime COPD symptoms and other outcomes, such as activities of daily living, health status, quality of life, and exacerbations; types of interventions used to treat daytime symptoms, including pharmacotherapy; and measures and tools used to assess daytime COPD symptoms
Study design	• All studies reporting any data on daytime COPD symptoms (e.g., multi- and single-center randomized controlled trials, nonrandomized trials, single-arm clinical trials, and real-world and observational studies) • Studies with full-text publications available only in languages other than English were excluded

*COPD* chronic obstructive pulmonary disease.

^a^Criteria were in line with Patient, Intervention, Comparator, Outcomes and Study Design (PICOS).

### Data extraction and analysis

Data relating to study design, patient population characteristics, and outcomes of interest were extracted from abstracts or full-text articles by a reviewer into a standardized data extraction form. Each form was then checked by two independent reviewers. Any discrepancies were resolved through discussion. Data from the final data extraction forms were tabulated and are discussed in this review in a purely descriptive fashion. Due to the heterogeneity of the data and the diversity of the clinical trial designs, no additional subanalyses or meta-analyses were planned or performed.

Terms such as “on waking”, “early morning”, “later in the morning”, and “morning” were all used in the identified articles; data related to these time points are discussed together in this review as “morning” symptoms, although the exact descriptions used in the source material are used wherever possible. With regards to descriptions of symptoms, the terminology used in the original source materials varied significantly; therefore, to simplify the reading of this review, standardized terms are used throughout. A glossary of these standardized terms is provided in Table 2.

**Table 2:** Glossary of standardized terms used in review article.

Standardized term	Alternative terms used in literature
Morning symptoms	• Symptoms on waking • Early morning symptoms • Symptoms later in the morning
Breathlessness	• Dyspnea (or dyspnea) • Shortness of breath
Cough	• Coughing • Increased cough • Persistent/recurring cough (±ing)
Increased sputum	• Expectoration • Phlegm (±production) • Phlegm/mucus • Excess phlegm •Increased sputum/mucus volume • Increased sputum/mucus production • Coughing up phlegm • Bringing up phlegm or mucus • Difficulty bringing up phlegm
Wheezing	• Increased wheezing • Wheezing or grunting
Chest tightness	• Tightness of the chest • Tightness in the chest

## Results

### Trial flow

**Fig. 1:**
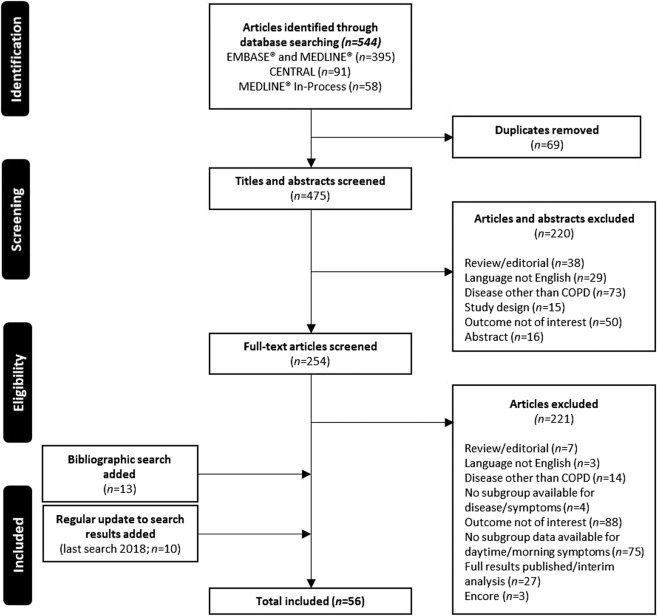
PRISMA trial flow diagram. COPD chronic obstructive pulmonary disease.

A total of 544 articles were identified by the initial searches (Fig. 1). After the first screening and removal of any duplicates, 255 full-text articles were selected for review. Following the second screening, 33 articles met the inclusion criteria. Thirteen additional articles eligible for inclusion were identified by manual searches of reference lists and bibliographies. An additional ten studies were included to capture literature published since the initial search, leading to a total of 56 articles.

### Trial characteristics and participants

Of the 56 articles included in the descriptive data analysis, 28 articles reported on randomized controlled trials, either in the form of primary data publication or in relation to subgroup, secondary, or pooled analyses. The remaining publications detailed result from a variety of studies, including cross-sectional, observational studies, noninterventional trials, survey- or interview-based assessments, or secondary analyses from existing real-world databases. Across the studies and analyses identified, data were presented on an estimated 40,000 patients with COPD (although some of these patients may have participated in more than one trial and/or analysis).

### Daytime COPD symptoms: prevalence, variability, and burden

Information on the prevalence (number of patients reporting symptoms) of common daytime symptoms of COPD was collated from 18 articles that had extractable data (Supplementary Table 4).^2,4,12,14–16,23–34^ In nine articles, the relative prevalence rates of COPD symptoms (both for all symptoms and for individual symptoms) were generally higher in the morning and/or daytime (when defined, this was usually the time between the morning period and when the patient goes to bed) compared with at night (Supplementary Table 4).^4,16,23,28–30,32–34^ For example, a prevalence of 81.4% for morning symptoms and 63.0% for night-time symptoms was reported by Miravitlles et al.^4^ Similar trends of morning and/or daytime versus night-time symptom prevalence were reported by Bateman et al.^23^ (94.4 versus 88.3%), Marth et al.^28^ (91.7 versus 70.6%), Soler-Cataluna et al.^29^ (71.3–83.5% versus 59.0–63.8%), Stephenson et al.^30^ (67.3 versus 50.0% of those completing the survey), Tsiligianni et al.^16^ (51.9 versus 39.4%), and Miravitlles et al.^33^ (71 versus 48%). Moreover, where prevalence rates were provided separately for morning and daytime, they were usually relatively similar.^4,26,29,32^ However, one study based on physician reports (not patient self-reporting) went against this trend.^15^ In this study by Roche et al., physicians reported morning symptoms for only 40% of their patients with COPD, compared with daytime symptoms for 97% of patients and night-time symptoms for 58% of patients. Breathlessness, cough, and increased sputum usually made up the top three most prevalent COPD symptoms reported for both morning and daytime, though relative proportions varied based on differences in populations and study designs (Supplementary Table 4).^4,12,14,23–27,29,30^ Wheezing and chest tightness/congestion were usually less commonly reported for morning and daytime.

Variability (variation in symptom prevalence across a 24-h period) of common daytime symptoms of COPD were reported in ten articles (Supplementary Table 4).^4,13–16,23,26,28–30^ Accumulated evidence from nine of these articles indicates that the prevalence of COPD symptoms tends to fluctuate over the course of the day.^4,14–16,23,26,28–30^ This is also demonstrated in a large study by Kessler et al.,^13^ where 44.7% of symptomatic patients perceived variability in one or more of their COPD symptoms throughout the day, with breathlessness and chest tightness varying the most. In three articles, the patients were asked about the variability of symptoms at different times of the day and night based on the perceived severity. Two articles found that a similar proportion of patients reported moderate to very severe symptoms in the morning, during the daytime, and at night.^4,30^ In the third study, more patients reported “at least moderate” symptoms in the morning than at night.^28^

A number of studies have described the fluctuation in symptomatic burden (bothersomeness and/or intensity of symptoms throughout the day) by asking respondents what time of day their symptoms were most troublesome, bothersome, distressing, or intense (a summary of the articles with extractable data is provided in Supplementary Table 5). Based on the combined findings of seven articles, the burden of COPD symptoms appears to be greatest in the morning.^12,13,17,25,27,35,36^ In a survey-based study by Decramer et al.,^35^ for example, 73% of reported respiratory symptoms were “most intense” in the morning, compared with 14% in the evening. In a large observational study, Partridge et al.^12^ similarly found that symptoms were “worse than usual” in the morning for 37% of study patients, versus 21% in the evening. Conversely, when Worth et al.^37^ analyzed data from almost 6000 patients entering the German DACCORD study, they found that a greater percentage of patients indicated symptoms were most bothersome during the daytime (56%) than in the morning (33%). In four articles where the day was broken down into shorter time categories (e.g., “morning”, “midday/noon”, “afternoon”, “evening”), there was a trend for the greatest symptom burden occurring in the morning period.^12,13,25,36^ Moreover, in the articles reporting on differences in symptom burden at different stages of the morning, there was a clear trend for the greatest impact occurring on waking, before tapering off slightly later in the morning.^13,25^ Partridge et al.^12^ reported that 37% of all the studied patients with COPD and 59% of patients with severe COPD were awoken by their symptoms in the morning at least 3 days a week.

Three of the analyzed articles reported on specific respiratory symptoms as an outcome:^13,25,27^ in all three, “morning” was the time of greatest burden from breathlessness, cough, and increased sputum, with a clear tapering off of these symptoms during the remainder of the day. Cough, for example, was reported by Kessler et al.^13^ as “most troublesome” upon waking/later in the morning in 49%/22% of study participants, compared with 15% in the afternoon and 19% in the evening. In the study by Kim et al.,^25^ cough was “most troublesome” upon waking/in the morning for 39%/33% of the patients who reported COPD symptoms, versus 16% in the afternoon and 7% in the evening; and in the Turkish COPD-SUNRISE observational study by Kuyucu et al.,^27^ 54% of participants reported cough as “most severe” in the morning, compared with 28% in the daytime. The trend for wheezing was less consistent among the identified studies. More patients in the studies by Kim et al.^25^ and Kessler et al.^13^ reported wheezing as “most troublesome” upon waking/later in the morning (62%/13% and 31%/22%, respectively), compared with the afternoon (17 and 18%) and evening (5 and 26%); however, in the Kuyucu et al.^27^ study, a similar proportion of patients reported wheezing as “most severe” in the daytime (33%) and in the morning (31%).^27^ Similar inconsistencies were seen for the pattern of burden for chest tightness. It is noteworthy, however, that when comparing the burden of symptoms at a specific time point, wheezing and chest tightness were usually the most burdensome symptoms in the afternoon, evening, and night, whereas breathlessness, cough, and increased sputum were usually the most burdensome symptoms on waking or in the morning (Supplementary Table 5).

### Daytime COPD symptoms: impact on other outcomes in daily life

In 13 articles, it was reported that daytime COPD symptoms had a negative impact on patients’ ability to perform normal daily activities (Supplementary Table 6).^4,12,13,15,17,24,25,27,28,30,35,36,38^ This was most notable in terms of morning activities, with five articles reporting that activities such as getting up/getting out of bed, personal hygiene (e.g., showering), and dressing were particularly impacted.^12,13,25,27,36^ For example, in a cross-sectional observational study of 472 COPD patients, Espinosa de los Monteros et al.^36^ found that 27% of patients had some type of impediment getting out of bed, and 29% and 33% had difficulties with personal hygiene and getting dressed, respectively. Moreover, morning or daytime “physical” activities (e.g., going up and down stairs, performing household chores, or doing exercise/sports) were also severely impacted.^12,13,17,24,27^ Although it was only reported in two articles related to survey-/interview-based studies, breathlessness was most commonly identified as the cause of patients’ reduced ability to perform normal daily activities.^12,17^

It is perhaps not surprising that in the studies where a relevant enquiry was made, a large proportion of patients indicated that they had altered their morning routine to accommodate their reduced ability to perform their normal activities, nor is it unexpected that patients ranked having well-controlled symptoms all day as the most important treatment attribute.^12,17,30,39^

Data from seven studies on the effects of daytime COPD symptoms on health status and/or QOL showed that there were raised levels of anxiety, depression, distress, and social inhibition/embarrassment among patients (Supplementary Table 6).^4,12,15,17,29,30,35^ Stephenson et al.,^30^ for example, noted that 54% of study patients with early morning symptoms reported feeling anxious (slightly to extremely), whereas O’Hagan and Chavannes^17^ found that 53% of surveyed participants had experienced social inhibition or embarrassment because of their morning symptoms. Of note, this relationship was based not only on subjective patient/physician reporting, but also on scores from validated measures, e.g., the COPD Assessment Tool (CAT), Clinical COPD Questionnaire (CCQ), Hospital, Anxiety and Depression Scale (HADS), and EuroQol Five Dimensions Questionnaire (EQ-5D). Articles from both Miravitlles et al. and Soler-Cataluna et al. reported significantly worse outcomes in terms of health status (CAT scores) and anxiety or depression (HADS scores) in patients with morning and daytime symptoms versus those without symptoms, whereas Roche et al. found that patients with morning symptoms had significantly worse CAT and EQ-5D scores than those without morning symptoms.^4,15^ Moreover, two articles reporting on the same study also noted a relationship between morning and daytime COPD symptoms and poor sleep quality, based on higher COPD and Asthma Sleep Impact Scale (CASIS) scores for patients with morning and daytime symptoms versus patients with no symptoms.^4,29^

In this review, four articles reported on the relationship between daytime COPD symptoms and exacerbations.^14–16,33^ In one, describing data from a large, cross-sectional, observational study, Tsiligianni et al.^16^ noted no statistically significant association between morning COPD symptoms and the likelihood of exacerbations 10–17 months after the baseline visit; this was based on logistic regression models. Similarly, in another large observational study, Miravitlles et al.^14^ showed that the presence of early morning or daytime symptoms were not independent predictors of exacerbations during the 6 months following baseline in a logistic regression model. Interestingly, in the two articles assessing the relationship between symptoms and a history of exacerbations (all based on large-scale, observational/real-world datasets), the presence of morning or daytime COPD symptoms was strongly associated with a higher frequency of exacerbations in the preceding 12 months.^14,15^ Another study by Miravitlles et al.^33^ found that any type of symptom variability was significantly associated with more exacerbations in the previous year.

The impact of COPD symptoms on other aspects of patients’ lives further emphasizes the overall burden of the disease. For example, O’Hagan and Chavannes^17^ noted that 63% of respondents in their study claimed that morning COPD symptoms had a negative impact on their working day. Roche et al. showed that patients in paid employment with morning symptoms had significantly more problems getting up and ready for the day, and had significantly more days off work in the 12 months prior to the study, compared with patients with COPD without morning symptoms. Miravitlles et al.^14^ have also shown significant relationships between both early morning and daytime symptoms and visits to the family doctor during a 6-month follow-up period, and between daytime COPD symptoms and visits to a specialist. Taken together, these results suggest that morning and daytime COPD symptoms could have serious financial implications for both the affected patient (through potential loss of earnings) and healthcare systems (through potential increased resource use).

### Daytime COPD symptoms: impact of interventions

Twenty-eight articles in this review looked specifically at the effects of interventions on daytime symptoms of COPD (Supplementary Table 7).^23,28,40–65^ These articles related to clinical studies of pharmacotherapy for COPD, and included evaluations of short-acting muscarinic antagonists (SAMA; ipratropium), long-acting muscarinic antagonists (LAMA; aclidinium, glycopyrronium, tiotropium), short-acting β_2_-agonists (SABA; albuterol, salbutamol), long-acting β_2_-agonists (LABA; indacaterol, formoterol, salmeterol), and inhaled corticosteroids (ICS; fluticasone, budesonide), either alone or in different combinations. Eighteen articles reported on placebo-controlled studies; 17 articles showed that the tested active therapies/combinations provided statistically significant improvements in daytime symptomatology compared with placebo^23,40,42,44,46–51,54,56–59,61,64^ and one study reported significant improvement in 24-h lung function with twice-daily LAMA/LABA compared with placebo.^65^ Combinations consisting of LAMA/LABA (±ICS) tended to improve daytime symptoms more effectively than either LABA or LAMA monotherapy.^23,54,56,59,61^ Many of these studies also reported benefits of pharmacotherapy on patients’ abilities to function normally, which generally manifested as an increase in the number of days that the patient could perform usual daily activities.^23,40,42,44,47,49–51,54,59,61^ One other article comprised a single-arm study of LAMA therapy in patients with COPD and reported significant reductions versus baseline in the severity of early morning symptoms, and hence in difficulties in performing morning activities.^28^ Eight articles looking at the impact of pharmacotherapy on daytime symptoms of COPD involved non-placebo-controlled head-to-head comparisons.^41,43,45,52,53,55,60,63^ Drug combinations including long-acting bronchodilators (LABA + ICS^41,43,52^ or LABA + SAMA^45^) were more effective than short-acting combinations (SABA + SAMA) at improving daytime symptom scores. In a study by Vogelmeier et al.,^60^ a LAMA/LABA combination significantly improved the number of symptom-free days and daytime breathlessness scores of patients with COPD when compared with a LABA + ICS combination; however, overall reductions in patient symptom scores were not significantly different between the two regimens. The findings of these pharmacologic studies are somewhat aligned with patient expectations of COPD treatments. Indeed, O’Hagan and Chavannes^17^ showed that 79% of patients agreed that their medication helps with their morning symptoms; more than half of patients stated that their medication helps their breathing, reduces shortness of breath, and improves their cough. However, only 21% said their medication improves their ability to carry out morning activities.

### Daytime COPD symptoms: measures and tools

The measures/tools used in the included articles varied widely (Supplementary Tables 4–5), and not all were validated for use in patients with COPD. The clinical trials we identified (Supplementary Table 7) tended to use a somewhat consistent selection of measures/tools. The validated Evaluating Respiratory Symptoms (E-RS) in COPD questionnaire was the most frequently used.^66^ Several articles described the development and validation of new measures/tools for specifically assessing daytime COPD symptoms. Mocarski et al.^67^ reported on the Early Morning Symptoms of COPD Instrument (EMSCI); Partridge et al.^68^ on the Capacity of Daily Living during the Morning (CDLM) questionnaire; Garrow et al.^69^ on the Manchester Early Morning Symptom Index (MEMSI); and Globe et al.^24^ on the COPD Morning Symptom Diary (COPD-MSD). One article explained the importance of a daily versus a weekly diary for capturing daily fluctuations in COPD symptoms.^70^ A further detailed analysis of the measures and tools used to assess the prevalence and burden of daytime symptoms, however, was beyond the scope of this literature review.

## Discussion

### Summary of key findings

This systematic review and literature analysis identified 56 articles reporting daytime COPD symptoms. Taken together, the accumulated evidence demonstrates that the symptomatic burden of COPD appears to be most acute in the morning, particularly at the time of waking. The prevalence of COPD symptoms tends to fluctuate over the course of the day, and is generally higher in the morning and/or daytime compared with at night. These morning symptoms have a substantial impact on patients’ ability to function normally (both in the morning and through the remainder of the day) and negatively affect patients’ QoL and health status. The literature confirms that pharmacotherapy can help patients to function normally during the day. Additionally, our analysis showed that the measures and tools used to evaluate daytime COPD symptoms are not uniform and vary widely across studies. We propose that there is need for a validated tool to better assess COPD symptoms, particularly morning symptoms.^71^

### Variability in daytime COPD symptoms

**Fig. 2:**
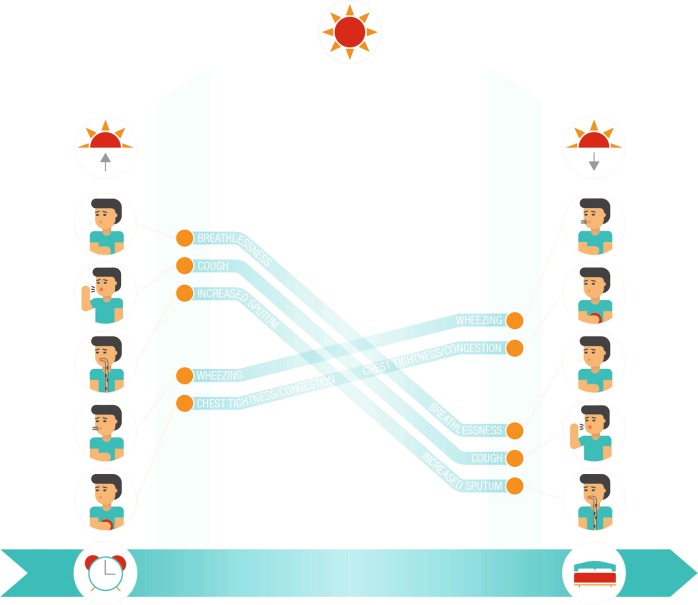
Daytime COPD symptoms.

The literature searches we conducted revealed a pattern of daytime symptomatology for patients with COPD. All characteristic symptoms (breathlessness, cough, increased sputum) and some of the associated symptoms (wheezing and chest tightness/congestion) are most prevalent and/or burdensome upon waking, with a gradual tapering off through the remainder of the morning and through lunchtime, before wheezing and chest tightness/congestion become prevalent and/or burdensome again in the afternoon or evening and into the night (Fig. 2). In another recent review, Singh et al.^72^ concluded that COPD symptoms are troublesome, variable, and can occur at any time during a 24-h period.

### Burden of daytime COPD symptoms

The literature showed a clear trend for the greatest burden from COPD occurring in the morning. This is perhaps an area that needs increased attention from both general practitioners and respiratory specialists. Indeed, Partridge et al.^12^ showed, via quantitative Internet interviews of >800 patients with COPD across multiple countries, that only 22–44% reported being asked by their physician whether they experience symptoms in the morning, and only 9–22% said they had been asked how they manage their morning routines. It should be noted that the article by Partridge et al. was published 10 years ago; however, there remains a strong argument for treating physicians to discuss with their patients with COPD when their symptoms are most troublesome and how this can be best managed.

### Negative impact of daytime COPD symptoms on QoL

This literature review showed that daytime COPD symptoms can negatively impact the lives of affected patients. This is demonstrated by the reduced ability to perform normal daily activities (e.g., going up and down stairs, household chores, shopping), as well as worsened QoL and health status (including increased anxiety/depression) and social embarrassment. It can also be surmised that, through their association with work-related issues (increased absenteeism) and increased medical resource use (higher numbers of physician/specialist/hospital visits), these daytime symptoms may also have the potential to cause a serious financial impact for both patients and healthcare services. These observations further emphasize the need for healthcare professionals to focus on patients’ morning symptoms and routines. Other support mechanisms (e.g., pulmonary rehabilitation programs) should also ensure that there is a similar specific focus on morning activities.

### Therapeutic options and their impact on daytime COPD symptoms

Many therapeutic options are available for the treatment of COPD, as identified in this literature review. They have the potential to mitigate daytime symptoms and can help patients function normally. Few studies specifically report on the effects of pharmacologic interventions on daytime or morning symptoms. Furthermore, O’Hagan and Chavannes^17^ showed that while the majority of patients agreed that medication improved their morning symptoms, this did not necessarily translate into an increased ability to carry out morning/daily activities. These observations point to a need for novel clinical studies that prospectively assess the various different medication classes, treatment combinations, and treatment regimens (e.g., once versus twice-daily) for their impact on daytime symptoms and the patients’ ability to function normally. The findings also support the continued search for newer interventions that may be even better suited to address this medical need. In addition, because the characteristic symptoms of COPD (breathlessness, cough, increased sputum) are particularly prevalent in the morning, future studies could also specifically assess which interventions may help patients manage these symptoms, and whether they are pharmacologic in nature or not (e.g., breathing exercises or pulmonary rehabilitation). It is argued that in order to truly ensure effective patient-orientated care in COPD, patients should be evaluated on the basis of lung function, frequency of symptoms, and the impact of symptoms on their QoL. Current COPD treatment decisions (selection of LAMA monotherapy, combination therapy with LAMA/LABA, or add-on therapy with LAMA to LABA/ICS) should be based on individualized assessment, ensuring personalization to the particular needs of the patient.^72,73^

### Tools and measures to assess daytime COPD symptoms

Many different tools have been used to measure and assess daytime symptoms of COPD, not all of which are validated for use in patients with the condition, or specific or focused enough to truly elicit the required information. The E-RS questionnaire, for example, is validated for use in COPD, and was used frequently in the identified clinical studies. This questionnaire is completed in the evening, and refers the end-user back to their experiences that “day”. However, depending on the end-user’s personal interpretation, that could mean they record the symptoms experienced since they awoke or, alternatively, the symptoms experienced since they completed the questionnaire the evening before (which would incorporate their night-time experiences). Similarly, one study reported on the prevalence of daytime sleepiness using results from the Epworth Sleepiness Scale.^2^ However, this scale is based upon a questionnaire that records the self-reported level of sleepiness; it does not collect information on the cause of that sleepiness.^74^ Indeed, since studies have commented on the close association between COPD and sleep disorders, such as insomnia and sleep apnea,^2,75^ the relationship between daytime COPD symptoms and daytime sleepiness is particularly complex. More focused and validated tools (e.g., the EMSCI, CDLM, MEMSI, and COPD-MSD) have been developed to specifically assess morning COPD symptoms and their secondary effects,^24,67–69^ but do not appear to be widely utilized to date. Additionally, there were two study design papers that describe the methodology of new studies in patients with COPD: STORICO, an observational study which aims to describe the frequency and evolution of early morning, daytime, and night-time COPD symptoms using clinically defined phenotypes; and FAntasTIGUE, a multicenter, longitudinal observational study which aims to assess severity and day-to-day/diurnal variations in fatigue.^76,77^ Once data are available, these studies will hopefully further help to determine frequency and changes in COPD-associated symptoms and fatigue.

### Strengths and limitations

The strengths of this systematic review include its broad search criteria and inclusive nature, with no date restrictions. Nevertheless, there are also several limitations that should be considered in relation to the interpretation of the findings. For example, for many of the identified articles, the data were extracted from observational studies or survey-/interview-based evaluations, which are inherently limited by their reliance on patient self-reporting. In some studies, symptom prevalence may not have been 100%, even though GOLD criteria for COPD diagnosis states that all patients must be symptomatic; this could again be attributable to patient self-reporting and variability in the type of survey questions.^1^ There was also considerable heterogeneity in multiple aspects of the study design, with no additional meta-analysis methodology used to account for this. In addition, as evidenced by the findings of this review, the manner in which daytime COPD symptoms were assessed varied widely, which could have influenced the trends and patterns observed. It is noteworthy, however, that these limitations relate to the evidence sources rather than the systematic review methodology.

Overall, the results of this study show that COPD symptoms are at their worst in the morning, particularly upon waking. These symptoms have a substantial impact on the ability of patients to function normally throughout the day, and they worsen patients’ quality of life. However, there are several treatments for COPD that can help reduce these symptoms. Physicians should initiate discussions with their patients regarding the time of day they suffer with symptoms the most, and adapt their clinical approach accordingly. In addition, the abundance of data reviewed suggests a need for: (1) a better understanding of how COPD symptoms (particularly morning symptoms) impact patient daily functioning, which could lead to better management approaches; (2) more studies specifically evaluating symptoms at well-defined periods throughout the day, using validated tools and measures; and (3) a consistent approach to measuring daytime symptoms and their impact on daily functioning.

## Supplementary information

Supplementary Information

## Data Availability

The data that support the findings of this study are publicly available through the following electronic biomedical literature databases: EMBASE®, MEDLINE®, MEDLINE® In-Process, and CENTRAL. Data sharing is not applicable to this article as no datasets were generated or analyzed during the current study.
