# Supplementary material for: Daytime symptoms of chronic obstructive pulmonary disease: a systematic review
Source: NPJ Prim Care Respir Med. 2020 Feb 21;30:6. doi: 10.1038/s41533-020-0163-5 (PMC7035364; doi:10.1038/s41533-020-0163-5)
Supplement: Supplementary file 1 — Supplementary Information [file 41533_2020_163_MOESM1_ESM.docx]

**Daytime symptoms of chronic obstructive pulmonary disease: a systematic review – ADDITIONAL FILE**

**Part 1: Search strategy**

# Supplementary Table 1. Search strategy for EMBASE® and MEDLINE® database searched via Embase.com

| No. | Query |
| --- | --- |
| 1 | “chronic obstructive lung disease”/exp OR “chronic obstructive lung disease”/syn OR “chronic airflow obstruction” OR “chronic airway obstruction” OR “chronic obstructive bronchitis” OR “chronic obstructive bronchopulmonary disease” OR “chronic obstructive lung disorder” OR “chronic obstructive pulmonary disease” OR “chronic obstructive pulmonary disorder” OR “chronic obstructive respiratory disease” OR “lung chronic obstructive disease” OR “lung disease, chronic obstructive” OR “lung diseases, obstructive” OR “obstructive lung disease” OR “obstructive lung disease, chronic” OR “obstructive pulmonary disease” OR “obstructive respiratory disease” OR “obstructive respiratory tract disease” OR “pulmonary disease, chronic obstructive” OR “pulmonary disorder, chronic obstructive” |
| 2 | “chronic obstructive lung disease”:ab,ti OR “copd”:ab,ti OR “chronic obstructive pulmonary disease” |
| 3 | #1 OR #2 |
| 4 | Symptom* NEAR/4 (“day” OR “daytime” OR “early morning” OR “morning” OR “afternoon” OR “24 hour”) |
| 5 | (“copd” OR “chronic obstructive pulmonary disease”) NEAR/2 (“daytime” OR “morning” OR “evening” OR “afternoon” OR “day”) |
| 6 | #3 AND #4 |
| 7 | #5 OR #6 |

# Supplementary Table 2. Search strategy for MEDLINE® In-Process, searched via PubMed.com interface

| No. | Query |
| --- | --- |
| 1 | “chronic obstructive pulmonary disease” [MeSH^®^ terms] |
| 2 | “copd” [Title/Abstract] |
| 3 | #1 OR #2 |
| 4 | (“symptom*” AND (“day” OR “daytime” OR “early morning” OR “morning” OR “afternoon” OR “24 hour”)) |
| 5 | (“daytime symptoms”) OR “symptom days” |
| 6 | #4 OR #5 |
| 7 | #3 AND #6 |
| 8 | ((“chronic obstructive pulmonary disease symptom*” OR “copd symptom*”) AND (“day” OR “daytime” OR “early morning” OR “morning” OR “afternoon” OR “24 hour”)) |
| 9 | #7 OR #8 |
| 10 | #9 AND (inprocess[sb] OR pubstatusaheadofprint) |

# Supplementary Table 3. Search strategy for CENTRAL searched via the Cochrane library interface

| No. | Query |
| --- | --- |
| 1 | MeSH^®^ descriptor: [Pulmonary Disease, Chronic Obstructive] explode all trees |
| 2 | “chronic obstructive pulmonary disease” OR “COPD” OR “chronic airflow obstruction” OR “chronic airway obstruction” OR “chronic obstructive bronchitis” OR “chronic obstructive bronchopulmonary disease” OR “chronic obstructive lung disorder” OR “chronic obstructive pulmonary disease” OR “chronic obstructive pulmonary disorder” OR “chronic obstructive respiratory disease” OR “lung chronic obstructive disease” OR “lung disease, chronic obstructive” OR “lung diseases, obstructive” OR “obstructive lung disease” OR “obstructive lung disease, chronic” OR “obstructive pulmonary disease” OR “obstructive respiratory disease” OR “obstructive respiratory tract disease” OR “pulmonary disease, chronic obstructive” OR “pulmonary disorder, chronic obstructive” |
| 3 | #1 or #2 |
| 4 | Symptom* NEAR/4 (“day” OR “daytime” OR “early morning” OR “morning” OR “afternoon” OR “24 hour”) |
| 5 | (“copd” OR “chronic obstructive pulmonary disease”) NEAR/3 (“daytime” OR “morning” OR “evening” OR “afternoon” OR “day”) |
| 6 | #3 AND #4 |
| 7 | #5 OR #6 |
| 8 | #7 (in trials) |

**Part 2: Literature analysis results**

**Supplementary Table 4. Prevalence of COPD symptoms throughout the day**

| **First author; year of publication** | **Study design** | **N** | **Measure/tool** | **Outcome** | **“Morning” symptoms (%)^#^** | **“Daytime” symptoms (%)^#^** |
| --- | --- | --- | --- | --- | --- | --- |
| Ali Zohal^1^; 2013 | Single-center, case-control study | 120 (COPD); 120 (controls) | Epworth Sleepiness Score (measures daytime sleepiness for any reason [i.e. not specifically related to daytime COPD symptoms]) | Daytime sleepiness | - | 31.6 (of patients with COPD) 15 (control group) |
| Bateman^2^; 2015 | Pooled analysis from two double-blind, randomized, parallel-group, active- and placebo-controlled, Phase III studies | 3,394 | Evaluating Respiratory Symptoms (E-RS) in COPD questionnaire  Early-Morning Symptoms of COPD Instrument (EMSCI) | Any symptom reported at baseline | 94.4 | - |
|  |  |  |  | Breathlessness | 77.6 | - |
|  |  |  |  | Cough | 81.5 | - |
|  |  |  |  | Difficulty bringing up phlegm | 48.6 | - |
|  |  |  |  | Wheezing | 57.4 | - |
| Globe^3^; 2016 | Qualitative interview-based study | 35 | COPD Morning Symptom Diary (COPD-MSD) | Breathlessness | 100.0 | - |
|  |  |  |  | Cough | 86 | - |
|  |  |  |  | Increased sputum | 89 | - |
|  |  |  |  | Wheezing | 69 | - |
|  |  |  |  | Chest tightness | 63 | - |
| Kim^4^; 2012 | Non-interventional, prospective, observational study | 133 | Clinical Symptoms Questionnaire (CSQ) | Any reported symptom | 57.1 | - |
|  |  |  |  | Breathlessness | 93.4 (of all patients reporting morning symptoms) | - |
|  |  |  |  | Cough | 69.7 (of all patients reporting morning symptoms) | - |
|  |  |  |  | Increased sputum | 85.1 (of all patients reporting morning symptoms) | - |
|  |  |  |  | Wheezing | 65.8 (of all patients reporting morning symptoms) | - |
|  |  |  |  | Chest tightness | 52.6 (of all patients reporting morning symptoms) | - |
| Kulich^5^; 2015 | Subset of patients from a multicenter, double-blind, parallel-group study | 209 | COPD eDiary | Any of five symptoms mentioned in eDiary (shortness of breath, phlegm/mucus, chest tightness, wheezing and coughing) | Lower frequency of all symptoms at morning assessment compared with evening assessment (not unexpected due to shorter assessment period) | |
| Kuyucu^6^; 2011 | National, multicenter, cross-sectional observational study | 514 | Data obtained directly from the patient, medical records, and physician in a single visit | Breathlessness | 47.9 | - |
|  |  |  |  | Cough | 25.1 | - |
|  |  |  |  | Increased sputum | 18.9 | - |
|  |  |  |  | Wheezing | 4.7 | - |
| Lu^7^; 2017 | Observational, cross-sectional, multi-site study | 1,025 (symptomatic patients) | Obtained directly from the patient during interview with physician | ≥1 symptom | 39.3 (on waking; of population experiencing symptom variability)  18.8 (later in the morning; of population experiencing symptom variability) | 7.9 (afternoon; of population experiencing symptom variability) |
| Marth^8^; 2015 | Multicenter, prospective, non-interventional study | 795 | Patient assessment using a  5-point Likert scale | Any early-morning COPD symptom reported at baseline | 91.7 | - |
|  |  |  |  | Breathlessness | 81.5 |  |
|  |  |  |  | Cough | 80.8 |  |
| Miravitlles^9, 10^; 2014, 2016 | Multinational, non-interventional, observational study | 727 | Self-administered 33-item Night-time, Morning and Daytime Symptoms of COPD questionnaire | ≥1 symptom in week prior to assessment  (% rated as moderate to very severe) | 81.4  (55.9 of all symptomatic patients who provided data for symptom severity) | 82.7  (56.9 of all symptomatic patients who provided data for symptom severity) |
|  |  |  |  | Breathlessness | 51.6 | 65.2 |
|  |  |  |  | Cough | 56.8 | 53.8 |
|  |  |  |  | Increased sputum | 52.4 | 44.6 |
|  |  |  |  | Wheezing | 28.7 | 29.2 |
|  |  |  |  | Chest tightness | 22.7 | 22.6 |
|  |  |  |  | Chest congestion | 16.8 | 14.3 |
| Miravitlles^11^; 2017 | Observational, prospective, multicenter study | 2,669 | Night-time and Early Morning Symptoms of COPD Instruments (NiSCI and EMSCI) | Any early morning symptom  (of which mild or moderately intense symptoms and intense and/or very intense symptoms) | 71  (95.7; 4.3) | - |
|  |  |  |  | Cough (early morning) | 56.8 | - |
|  |  |  |  | Wheezing | 18.8 | - |
|  |  |  |  | Shortness of breath | 39.2 | - |
|  |  |  |  | Chest tightness | 12.8 | - |
|  |  |  |  | Chest congestion | 12.9 | - |
|  |  |  |  | Difficulty expulsing phlegm | 29.3 | - |
| Munoz^12^; 2018 | Multinational real-world study | 8,185 | Cross-sectional survey of patients with COPD | Physician-reported symptoms | 4 (morning only)  17 (morning/day only) | 16 (day only) |
| Partridge^13^; 2009 | Internet-based, interview study | 289 (cohort of patients with severe COPD) | Internet interviews using a 34-item questionnaire | Breathlessness | 78 (of severe COPD cohort) | - |
|  |  |  |  | Cough | 60 (of severe COPD cohort) | - |
|  |  |  |  | Increased sputum | 63 (of severe COPD cohort) | - |
| Roche^14^; 2013 | Analysis of data from a large, multinational, cross-sectional, survey-based study | 1,489 | Patient record form (PRF) completed by physician | Physician-reported symptoms | 40 | 97 |
|  |  |  |  | Breathlessness (when exercising) | 9 | 74 |
|  |  |  |  | Breathlessness (at rest) | 6 | - |
|  |  |  |  | Cough | 27 | 72 |
|  |  |  |  | Increased sputum | 21 | - |
|  |  |  |  | Chest tightness | 6 | - |
|  |  |  |  | Wheezing | 8 | - |
| Soler-Cataluña^15^; 2016 | Multinational,  non-interventional, observational study | Spain (S)  122  Other countries (O)  605 | Self-administered 33-item Night-time, Morning and Daytime Symptoms of COPD questionnaire  Prevalence data for individual symptoms only reported for Spanish cohort | ≥1 symptom in week prior to assessment | 71.3 (S)  83.5 (O) | 71.3 (S)  85.0 (O) |
|  |  |  |  | Breathlessness | 29.5 (S) | 46.7 (S) |
|  |  |  |  | Cough | 51.6 (S) | 45.9 (S) |
|  |  |  |  | Increased sputum | 50.8 (S) | 46.7 (S) |
|  |  |  |  | Wheezing | 17.2 (S) | 18.0 (S) |
|  |  |  |  | Chest tightness | 12.3 (S) | 11.5 (S) |
|  |  |  |  | Chest congestion | 10.7 (S) | 8.2 (S) |
| Stephenson^16^; 2015 | Cross-sectional patient survey linked with medical claims data | 752 | Telephone-based survey | Responders with early morning symptoms | 67.3 (of respondents who completed the survey) | - |
|  |  |  |  | Breathlessness | 73.4 (of respondents with early morning symptoms) | - |
|  |  |  |  | Cough | 74.5 (of respondents with early morning symptoms) | - |
|  |  |  |  | Increased sputum | 69.6 (of respondents with early morning symptoms) | - |
|  |  |  |  | Wheezing | 59.3 (of respondents with early morning symptoms) | - |
|  |  |  |  | Chest tightness | 40.9 (of respondents with early morning symptoms) | - |
|  |  |  |  | Chest congestion | 46.1 (of respondents with early morning symptoms) | - |
| Tsiligianni^17^; 2016 | Cross-sectional, longitudinal, observational study | 2,269 | Asthma Control Questionnaire (ACQ) | Any symptom | 51.9 | - |
|  |  |  |  | Severe symptoms | 4.9 | - |
| van Buul^18^; 2017 | Single-center, observational, cross-sectional study | 80 | PRO-Morning COPD Symptoms Questionnaire | Any morning symptom | 96 | - |

^#^: % of total population unless otherwise stated.

COPD: chronic obstructive pulmonary disease; O: other (non-Spanish) European population; S: Spanish population.

**Supplementary Table 5. Burden of COPD symptoms throughout the day**

| **First author; year of publication** | **Type of study** | **N** | **Measure/tool** | **Outcome** | **“Morning” symptoms (%)^#^** | **“Daytime” symptoms (%)^#^** | **“Midday”/ “Noon” symptoms (%)^#^** | **“Afternoon” symptoms (%)^#^** | **“Evening” symptoms (%)^#^** |
| --- | --- | --- | --- | --- | --- | --- | --- | --- | --- |
| Decramer^19^; 2013 | Survey-based study | 116 | Structured questionnaire completed by pulmonary physicians | **When were your patients’ symptoms “most intense”?**  Note: Data shown are % of reported symptoms | | | | | |
|  |  |  |  | Respiratory symptoms (included cough with expectoration, dyspnea, frequent or persistent cough, wheezing, and tightness) | 73 | - | 8 | 8 | 14 |
|  |  |  |  | Other symptoms (included depression/ anxiety, loss of appetite, fatigue and others) | 23 | - | 4 | 18 | 27 |
| Espinosa de los Monteros^20^; 2012 | Multinational, cross-sectional, observational study | 472 | Observational, epidemiologic, cross-sectional study; patients were interviewed by telephone | **Periods of the day in which respiratory symptoms were more acute (in patients who declared that one or more of their symptoms varied throughout the day; *n* = 152)**  Note: Data estimated from graph and are approximate values | | | | | |
|  |  |  |  | Dyspnea | 44 | **-** | 16 | 16 | **-** |
|  |  |  |  | Expectoration | 70 |  | 14 | 11 |  |
|  |  |  |  | Cough | 58 |  | 15 | 13 |  |
|  |  |  |  | Wheezing | 48 |  | 15 | 16 |  |
|  |  |  |  | Tightness | 36 |  | 32 | 23 |  |
| Kessler^21^; 2011 | Multinational, cross-sectional, observational study | 2,441 | Telephone-based interviews (patient questionnaires) | **During what time of day is the symptom “most troublesome”?**  Note: Values given in “morning” column represent “on waking” and “later in the morning” categories, respectively; values in parentheses are proportions of patients who reported most troublesome symptoms upon waking and/or later in the morning. Percentages shown are based on various denominators related to the number of patients experiencing each symptom.  Similar outcomes were reported by Espinosa de los Monteros *et al.* for the Spanish cohort from the same study.^20^ | | | | | |
|  |  |  |  | Breathlessness (*n*= 1,769) | 31.0 / 24.0 (45.4) | - | - | 22.5 | 19.5 |
|  |  |  |  | Cough (*n*= 1,433) | 48.9 / 22.3 (60.1) | - | - | 14.9 | 18.7 |
|  |  |  |  | Increased sputum (*n*= 1,551) | 56.7 / 26.2 (70.9) | - | - | 16.3 | 16.6 |
|  |  |  |  | Wheezing (*n*= 1,018) | 31.1 / 21.7 (43.4) | - | - | 18.3 | 26.1 |
|  |  |  |  | Chest tightness (*n*= 690) | 28.8 / 25.9 (45.4) | - | - | 25.4 | 25.5 |
| Kim^4^; 2012 | Non-interventional, prospective, observational study | 133 | Clinical Symptoms Questionnaire (CSQ) | **When were your symptoms the “most troublesome” for you?**  Note: Values given in “morning” column represent “on waking” and “in the morning” categories, respectively. | | | | | |
|  |  |  |  | Breathlessness | 63.6 / 21.5 (of patients who reported COPD symptoms) | - | - | 11.6  (of patients who reported COPD symptoms) | 1.7  (of patients who reported COPD symptoms) |
|  |  |  |  | Cough | 39.3 / 33.3 (of patients who reported COPD symptoms) | - | - | 15.5  (of patients who reported COPD symptoms) | 7.1  (of patients who reported COPD symptoms) |
|  |  |  |  | Increased sputum | 35.1 / 52.1 (of patients who reported COPD symptoms) | - | - | 6.4  (of patients who reported COPD symptoms) | 2.1  (of patients who reported COPD symptoms) |
|  |  |  |  | Wheezing | 62 / 13.4  (of patients who reported COPD symptoms) | - | - | 16.5  (of patients who reported COPD symptoms) | 5.1  (of patients who reported COPD symptoms) |
|  |  |  |  | Chest tightness | 54.5 / 20  (of patients who reported COPD symptoms) | - | - | 16.4  (of patients who reported COPD symptoms) | 1.8  (of patients who reported COPD symptoms) |
| Kuyucu^6^; 2011 | National, multicenter, cross-sectional observational study | 514 | Data obtained directly from the patient, medical records and physician in a single visit | **When was the symptom “variability most severe”?** | | | | | |
|  |  |  |  | Breathlessness (*n*= 509) | 41.1 | 39.7 (*p*< 0.001 versus morning) | - | - | - |
|  |  |  |  | Cough (*n*= 473) | 53.5 | 27.5 (*p*< 0.001 versus morning) | - | - | - |
|  |  |  |  | Increased sputum (*n*= 477) | 61.0 | 30.0 *(p*< 0.001 versus morning) | - | - | - |
|  |  |  |  | Wheezing (*n*= 465) | 31.2 *(p*< 0.001 versus night-time) | 32.9 *(p*< 0.001 versus night-time) | - | - | - |
|  |  |  |  | Chest tightness (*n*= 421) | 26.4 (*p*< 0.001 versus daytime) | 52.3 | - | - | - |
| O’Hagan^22^; 2013 | Survey-based study | 811 | Structured questionnaire completed online by patients | **Symptoms of COPD that were worse in the morning** | | | | | |
|  |  |  |  | Cough | 57 | **-** | **-** | **-** |  |
|  |  |  |  | Shortness of breath | 46 |  |  |  |  |
|  |  |  |  | Coughing up phlegm | 43 |  |  |  |  |
| Partridge^13^; 2009 | Quantitative, Internet-based, interview-based study | All (A)  803  Severe cohort (SC)  289 | Internet interviews using a 34-item questionnaire | **When were your symptoms “worse than usual”?** (multiple answers were possible) | | | | | |
|  |  |  |  | All COPD symptoms | 37 (A) (*p*< 0.001 versus midday, afternoon, evening and night groups) | - | 4 (A) | 9 (A) | 21 (A) |
|  |  |  |  |  | 46 (SC) *(p*< 0.001 versus midday) | - | 11 (SC) | 16 (SC) | 27 (SC) |
| Worth^23^; 2016 | Longitudinal, prospective non-interventional study | 5,924 | Patient-reported symptoms on entry to DACCORD study | **What time of day were your symptoms “most bothersome”?** | | | | | |
|  |  |  |  | COPD symptoms (symptoms reported were: exertional dyspnea, dyspnea at rest, chest tightness/chest pain, cough, wheezing or grunting, prolonged expiration, restricted exercise tolerance) | 33.2 (of total population, data missing from *n*= 32) | 55.9 (of total population; data missing from *n*= 32) | - | - | 3.6 (of total population; data missing from *n*= 32) |

^#^: % of total population unless otherwise stated.

A: complete cohort; COPD: chronic obstructive pulmonary disease; SC: severe cohort.

**Supplementary Table 6. Relationship between daytime COPD symptoms and normal daily activities, health status and QoL**

| **First author & reference; year of publication** | **Type of study** | **N** | **Impact on normal daily activity** | **Impact on health status/QoL** |
| --- | --- | --- | --- | --- |
| Decramer^19^; 2013 | Survey-based study | 116 | - 89% of surveyed physicians declared that variability of symptoms during the day impacted the mobility of the patients - 84% of physicians believed that impairments in mobility occurred mainly in the morning and much less at other times of the day (16% started at noon, 9% in the afternoon, 6% in the evening, and 5% at night) | - 97% of surveyed physicians declared that the variability of symptoms during the day affected the patients’ QoL |
| Espinosa de los Monteros^20^; 2012 | Multinational, cross-sectional, observational study | 472 | - When asked about the impact of the symptoms on morning activities, 26.5% had some type of impediment to get out of bed, 28.8% had difficulties during personal hygiene, and 32.8% had difficulty getting dressed | *Not reported* |
| Globe^3^; 2016 | Qualitative interview-based study | 35 | - Almost all subjects (*n* = 33) spontaneously discussed one or more common indoor morning activity or routine that would trigger their COPD symptoms (e.g. going up and down stairs) | *Not reported* |
| Kessler^21^; 2011 | Multinational, cross-sectional, observational study | 2,441 | - Morning activities that patients felt were most affected by their COPD symptoms were washing (41.0%), dressing (40.7%), drying (36.2%), and getting out of bed (35.4%) - 9.5% (*n* = 231) of the patient population reported that they required assistance to perform their normal morning activities and, of these patients, 67.5% (*n* = 156) felt worried they were a burden to other people as a result of their limited activities - Daily activities that patients felt were most affected by COPD symptoms were going up and down stairs (82.5%), doing heavy household chores (56.9%), going shopping (43.1%) and doing sport or hobbies (35.9%) - 9.4% of respondents were unable to exercise outside | *Not reported* |
| Kim^4^; 2012 | Non-interventional, prospective, observational study | 133 | - Of the morning activities limited by morning symptoms, the most frequently reported was getting out of bed (82.9%), followed by using the toilet (77.6%), drying (77.6%), washing yourself (76.3%), and dressing yourself (69.7%) - Washing yourself was the morning activity most severely limited by COPD symptoms | *Not reported* |
| Kuyucu^6^; 2011 | National, multicenter, cross-sectional observational study | 514 | - Most commonly affected morning activity was climbing up/down the stairs (point of effect: 6.7), followed by wearing socks/shoes (point of effect: 4.3) and showering/bathing (point of effect: 4.2) | *Not reported* |
| Marth^8^; 2015 | Multicenter, prospective, non-interventional study | 795 | - At the baseline visit, 49.9% (*n* = 397) of patients were at least moderately impaired in the performance of their morning activities due to COPD, with a further 37.5% (*n* = 298) being mildly impaired | *Not reported* |
| Miravitlles^10^; 2014 | Multinational, non-interventional, observational study | 727 | - 64.2% of patients in the overall population who were categorized as “sedentary” had symptoms throughout the whole 24-h day compared with 50.4% of patients categorized as “active” - In each part of the 24-h day, there was a significant relationship between symptoms and patients’ physical activity level at baseline (*p*< 0.05 for each part of the day) | - Early morning and daytime symptoms were significantly associated with worse outcomes on measures of health status (CAT scores), anxiety/depression (HADS scores), and sleep quality (CASIS scores) versus patients without symptoms over same time period; *p*< 0.001 - Outcomes were worse among patients experiencing symptoms during both time periods versus only one time period |
| Miravitlles^11^; 2017 | Observational, prospective, multicenter study | 2,669 | *Not reported* | - Combined COPD symptom variability (reflecting variability in symptom number or intensity), which is experienced more in the morning than at night, was associated with worse dyspnea, with more exacerbations in the previous year, and with an exacerbator or asthma-COPD overlap phenotype - Variability in the intensity of symptoms was associated with anxiety |
| O’Hagan^22^; 2014 | Survey-based study | 811 | - The proportion of patients reporting an “extreme impact/I am no longer able to do this activity” for “going up and down stairs” was 20% and for “doing morning chores around the house” was 15% - “Shortness of breath” was identified as the commonest cause of reduced ability to perform a daily task - All daily activities took longer to complete than before the patients began experiencing worse COPD symptoms in the morning; for example, “getting up” took an average 12 min longer, “doing morning chores around the house” took an average 32 min longer - 49% of patients said they had had to make changes to their morning routine due to their morning COPD symptoms - 54% said the symptoms suffered in the morning continued to impact routines throughout the day | - 53% confirmed that they had suffered social inhibition or embarrassment at some point due to their morning COPD symptoms |
| Partridge^13^; 2009 | Quantitative, Internet-based, interview-based study | 803 | - 74% of all patients with COPD and 96% of patients with severe COPD reported that they took longer to complete their morning routine than they used to, with 48% of all patients with COPD and 84% of patients with severe COPD adapting the way they did some or all of their activities - Based on a scale of 1 to 10 (with 1 = not affected at all and 10 = greatly affected), the morning activities most affected by COPD were: “walking up and down stairs” (6.2 for all COPD and 8.6 for severe COPD); “putting on shoes and socks” (4.4 for all COPD and 6.7 for severe COPD); and “making the bed” (4.3 for all COPD and 6.8 for severe COPD) - 52% of patients received help with making the bed and 46% with washing the dishes - Shortness of breath was the symptom most strongly correlated with the extent of problems experienced with the morning routine | - 37% of all patients with COPD and 73% of patients with severe COPD regarded problems associated with morning routines as bothersome - 19% of patients with severe COPD considered the problems with their morning routine to have a very strong negative impact on their QoL and general well-being; a further 21% of patients with severe COPD felt the morning routine problems bothered them considerably |
| Roche^14^; 2013 | Analysis of data from a large, multinational, cross-sectional, survey-based study | 1,489 | - Impact on normal daily activities, measured on a 7-point Likert scale of no impact to constant impact (where 7 = constant impact) was significantly higher in those with morning symptoms compared with those without (3.96 vs. 3.29, respectively) | - Patients with morning symptoms had significantly worse CAT and EQ-5D scores than those without morning symptoms, indicating a negative impact on health status and QoL - In a multivariate model, morning symptoms were significantly associated with a physician-confirmed diagnosis of depression (*p*= 0.021) |
| Soler-Cataluña^15^; 2016 | Multinational,  non-interventional, observational study | 122 | *Not reported* | - Morning and daytime symptoms were significantly associated with worse outcomes on measures of health status (CAT scores), depression (HADS depression subscale scores), and sleep quality (CASIS scores) (*p*< 0.05) |
| Stephenson^16^; 2015 | Cross-sectional patient survey linked with medical claims data | 752 | - 60.4% reported limiting their morning activities due to early morning symptoms, and 27.8% had trouble concentrating in the morning | - 54.3% with early morning symptoms reported feeling anxious (slightly to extreme) |
| Svedsater^24^; 2017 | Internet-based survey | 302 | - When patients with COPD ranked treatment attributes on a scale of 0–8, with the lowest scores assigned to the most important, the mean rankings were (in decreasing order of importance): well-controlled symptoms (3.11); frequency of flare ups/exacerbation (3.79); physical activities (3.82); medication frequency (4.15); sleep disturbance (4.53); social activities (4.59); inhaler ease of use and convenience (4.87); and monthly cost (7.14) | *Not reported* |
| Tabak^25^; 2012 | Telemonitoring study | 39 | - Mean daily activity in patients with COPD was significantly lower compared with healthy controls (864±277 cpm vs. 1,162±282 cpm; *p*< 0.001) | *Not reported* |
| Tsiligianni^17^; 2016 | Cross-sectional, longitudinal, observational study | 2,269 | *Not reported* | - Patients with morning or night-time symptoms had worse health status outcomes versus those without morning or night-time symptoms (CCQ scores) - Presence of moderate (*p*= 0.006) or severe (*p* < 0.000) morning symptoms was significantly associated with a poor health status (based on a CCQ score ≥1, 10–17 months after baseline visit) |
| van Buul^18^; 2017 | Single-center, observational, cross-sectional study (Morning symptoms in-Depth observAtional Study [MODAS]) | 80 | - Morning symptom severity was associated with lower physical activity (measured by accelerometry): fewer steps a day (estimated regression coefficient = -0.001, 95% CI -0.002 to -0.000), less time in moderate to vigorous physical activity with bouts of at least 10 minutes (estimated regression coefficient = -0.135, 95% CI -0.233 to -0.037); and less time in moderate physical activity with bouts of at least 10 minutes (estimated regression coefficient = -0.192, 95% CI -0.321 to -0.064) | - Morning symptom severity was associated with lower health status (estimated regression coefficient = 1.194, 95% CI 0.923 to 1.465), higher symptomatic burden (estimated regression coefficient = 4.193, 95% CI 2.384 to 6.002), increased anxiety and depression, and lower FEV_1_ |
| van Buul^26^; 2018 | Single-center, observational, cross-sectional study (Morning symptoms in-Depth observAtional Study [MODAS]) | 79 | - Mean (±SD) number of steps per day was 5,686±3,514. - Patients with low morning symptom scores took 6,598±4,243 steps a day; those with high morning symptom scores 4,727±2,209 steps a day (mean difference 1,871, *p*= 0.017). - Patients with high morning symptom scores took significantly fewer steps during the morning (mean difference 669, *p*= 0.030) and during the afternoon (mean difference 1,013, *p*= 0.015) than patients with low morning symptom scores | *Not reported* |

CASIS: COPD and Asthma Sleep Impact Scale; CAT: COPD Assessment Tool; CCQ: Clinical COPD Questionnaire; COPD: chronic obstructive pulmonary disease; cpm: counts per minute; EQ-5D: EuroQol Five Dimensions Questionnaire; FEV_1_: forced expiratory volume in 1 second; HADS: Hospital, Anxiety and Depression Scale; QoL: quality of life.

# Supplementary Table 7. Summary of identified articles describing the effects of interventions on daytime COPD symptoms

| **First author & reference; publication year** | **N** | **Relevant intervention(s)** | **Relevant measures** | **Overview of relevant outcomes** |
| --- | --- | --- | --- | --- |
| Bateman^2^; 2015 | 3,394 | 1. Aclidinium/formoterol 400/12 µg BID 2. Aclidinium 400 µg BID 3. Formoterol 12 µg BID 4. Placebo | Early-morning symptoms of COPD instrument (EMSCI)  COPD symptoms questionnaire (via eDiary)  Evaluating Respiratory Symptoms (E-RS) questionnaire^#^ | Over 24 weeks, aclidinium/formoterol significantly improved early-morning symptom severity compared with placebo, including both overall and individual symptom severity scores (cough, wheezing, shortness of breath, and difficulty bringing up phlegm) (*p* < 0.05). The change in overall early-morning symptom severity scores was also significantly greater with the combination therapy versus each monotherapy (*p* < 0.01)  Compared with placebo, aclidinium/formoterol significantly improved limitation of activities due to morning symptoms (*p* < 0.001)  Over the 24 weeks, E-RS total score was significantly improved with aclidinium/formoterol compared with placebo and both monotherapies (*p* < 0.001 versus placebo; *p* < 0.01 versus both monotherapies) |
| Beier^27^; 2013 | 414 | 1. Aclidinium 400 μg BID 2. Tiotropium 18 μg QD 3. Placebo | COPD symptoms questionnaire (via eDiary)  E-RS | Severity of early-morning symptoms significantly reduced over 6 weeks with aclidinium (*p* < 0.001) and tiotropium (*p* < 0.05) versus placebo; there was no difference between the active treatments  E-RS total scores were significantly reduced from baseline with both aclidinium (*p* < 0.0001) and tiotropium (*p* < 0.05) versus placebo  Limitation of activity caused by COPD symptoms was also significantly reduced from baseline over 6 weeks with aclidinium versus placebo but not with tiotropium versus placebo (*p* < 0.05 for aclidinium versus tiotropium) |
| Beier^28^; 2017 | 277 | 1. Aclidinium bromide 400 µg BID 2. Tiotropium 18 µg QD 3. Placebo | Evaluating Respiratory Symptoms (E-RS) questionnaire^#^  COPD symptoms questionnaire (via eDiary) | Aclidinium 400 µg BID provided additional improvements compared with tiotropium 18 µg QD in: (1) bronchodilation (2) daily COPD symptoms (E-RS), (3) early-morning symptoms and (4) early-morning limitation of activity. |
| Bleecker^29^; 2008 | 719 | 1. Ipratropium bromide/albuterol 36/206 µg QID 2. Fluticasone propionate/salmeterol 250/50 µg BID (FSC) | Patient daily diary cards | In patient cohorts based on β-agonist reversibility or non-reversibility, the overall daytime symptom score over Weeks 1–8 was reduced by 21% and 22%, respectively, with FSC, and by 14% and 14%, respectively, with ipratropium bromide/albuterol. In both the reversible and non-reversible groups, the reduction in overall daytime symptom score was significantly larger with FSC compared with ipratropium bromide/albuterol (*p =*0.044 at endpoint) |
| Dahl^30^; 2013 | 339 | 1. QVA149 (110 µg indacaterol/50 µg glycopyrronium) 2. Placebo | Patient diary | Symptom scores improved from baseline significantly more with QVA149 than placebo (daytime symptom score, *p=*0.030)  Over 52 weeks, QVA149 was also significantly better than placebo for percentage of days with “no daytime symptoms” (*p=*0.012) and percentage of “days able to perform usual daily activities” (*p=*0.028) |
| Donohue^31^; 2004 | 365 | 1. Ipratropium bromide/albuterol 36/206 µg QID 2. Fluticasone propionate/salmeterol 250/50 µg BID (FSC) | Patient daily diary cards | In both groups, progressive improvement in daytime symptom scores was observed over the treatment period with significantly greater improvements observed with FSC than with ipratropium bromide/albuterol (*p* ≤ 0.044) |
| Donohue^32^; 2010 | 2,059 | 1. Indacaterol 150 µg 2. Indacaterol 300 µg 3. Tiotropium 18 µg (open-label) 4. Placebo | Patient daily diary cards | The percentage of days with no daytime symptoms was increased for indacaterol 150 or 300 µg versus placebo (*p* < 0.05), but was not significantly different for tiotropium versus placebo  The percentage of days able to perform usual activities was increased for indacaterol 150 or 300 µg (*p* < 0.001) and tiotropium (*p* < 0.05) versus placebo |
| D’Urzo^33^; 2001 | 159 | 1. Formoterol 12 µg BID/ipratropium 40 µg QID 2. Salbutamol 200 µg QID /ipratropium 40 µg QID | Daily patient diary | All mean individual symptom scores were lower with formoterol/ipratropium than with salbutamol/ipratropium; moreover, the mean total daily symptom score was significantly lower with formoterol/ipratropium than with salbutamol/ipratropium (*p =*0.0042) |
| D’Urzo^34^; 2014 | 1,692 | 1. Aclidinium/formoterol 400/12 µg BID 2. Aclidinium/formoterol 400/6 µg BID 3. Aclidinium 400 µg BID 4. Formoterol 12 µg BID 5. Placebo | EMSCI  E-RS questionnaire | The average rating for overall early-morning COPD symptom severity via the EMSCI was significantly improved for the aclidinium/formoterol combinations compared with placebo at all timepoints including study end (*p* < 0.01)  Over 24 weeks, significant improvements in overall average daily E-RS scores were observed with both combinations and both monotherapies compared with placebo (*p* < 0.01) |
| D’Urzo^35^; 2014 | 1,854 | 1. Glycopyrronium 50 µg 2. Tiotropium 18 µg (open-label) 3. Placebo | Patient daily diary cards | Glycopyrronium and tiotropium significantly improved the change from baseline for all total and almost all individual symptom scores over the 12-, 26- and 52-week treatment period compared with placebo  Glycopyrronium demonstrated a significant increase in the percentage of days with “no daytime symptoms” versus placebo at Week 52 (*p =*0.039) and a numerical increase at Week 26. Glycopyrronium also provided a significant increase in the percentage of “days able to perform usual activities” versus placebo at Week 26 (*p =*0.014), and a numerical increase at Week 52 (*p =*0.399) *NOTE: data were not provided for tiotropium* |
| Jones^36^; 2016 | 1,161 | 1. Aclidinium 400 μg BID 2. Placebo | E-RS questionnaire | After 24 weeks, in the overall patient population, the improvements from baseline in E-RS total score and individual domain scores were significantly greater with aclidinium compared with placebo (*p* < 0.001)  The net benefit in E-RS total score was significantly higher in patients treated with aclidinium than in those receiving placebo (*p* < 0.01) |
| Kerwin^37^; 2012 | 561 | 1. Aclidinium 400 µg BID 2. Aclidinium 200 µg BID 3. Placebo | Night-time symptoms questionnaire^¶^ | Compared with placebo at Week 12, both aclidinium doses significantly reduced the severity of breathlessness in the first hour after waking up and the impact of breathlessness on early-morning activity (*p* < 0.01) |
| Kostikas^38^; 2018 | 4,389 | 1. Indacaterol/glycopyrronium 2. Glycopyrronium | eDiary | Early symptom improvement after treatment with indacaterol/glycopyrronium at Day 7 or 14, may identify patients with clinically important improvement in lung function and dyspnea at Week 12 |
| LaForce^39^; 2016 | 441 | 1. Glycopyrrolate 15.6 µg BID 2. Placebo | eDiary  COPD Assessment Test (CAT) | Glycopyrrolate significantly improved daily total symptom score and daytime total symptom score versus placebo (*p* < 0.05). The percentage of days able to perform usual daily activities was also significantly different in favor of glycopyrrolate (*p* < 0.01)  Decrease in CAT score from baseline was significant (*p =*0.040) in favor of glycopyrrolate |
| Magnussen^40^; 2010 | 96 | 1. Indacaterol 300 µg pm (8–11 pm) 2. Indacaterol 300 µg am (8–11 am) 3. Salmeterol 50 µg BID 4. Placebo | eDiary | Over 14 days of treatment, compared with placebo, both indacaterol regimens significantly improved the percentage of days with no daytime symptoms and the percentage of days able to perform usual activities (*p* < 0.05)  Improvements in all of these analyses were consistently in favor of indacaterol (both regimens) over salmeterol. There were no significant differences between the two indacaterol regimens |
| Make^41^; 2005 | 361 | 1. Ipratropium bromide/albuterol 36/206 µg QID 2. Fluticasone propionate/salmeterol 250/50 µg BID (FSC) | Patient diary cards | A significantly greater reduction in the overall daytime symptom score from Weeks 1–8 was observed for patients treated with FSC compared with ipratropium bromide/albuterol (*p =*0.024). At endpoint, both treatments resulted in a reduction in the overall daytime symptom score, but the difference between treatments was not significant |
| Marin^42^; 2016 | 124 | 1. Glycopyrronium 50 µg 2. Tiotropium 18 µg   [Cross-over design] | PRO – Morning COPD Symptoms Questionnaire^+^ | Glycopyrronium and tiotropium improved morning symptom scores similarly post-dose at Day 1 and Day 28  Glycopyrronium, but not tiotropium, showed superior improvement in pre-dose score versus baseline after 4 weeks (*post hoc* analysis) |
| Marth^8^; 2015 | 795 | 1. Aclidinium 400 µg BID (via Eklira Genuair inhaler) | Patient interviews | There were significant reductions in the severity of early-morning symptoms and the limitation of morning activities in patients under treatment with the Eklira Genuair inhaler |
| McGarvey^43^; 2016 | 1,792 | 1. Aclidinium 200 µg or 400 µg BID 2. Tiotropium 18 µg QD 3. Placebo | E-RS questionnaire^#^  COPD symptoms questionnaire (via eDiary) | Improvements were seen with aclidinium versus placebo in severity of morning cough and phlegm |
| Miravitlles^44^; 2016 | 3,394 | 1. Aclidinium/formoterol 400/12 µg BID 2. Aclidinium 400 µg BID 3. Formoterol 12 µg BID 4. Placebo | COPD symptoms questionnaire (via eDiary)  E-RS questionnaire | Aclidinium/formoterol reduced early-morning symptom severity from baseline in more symptomatic patients at Week 24 compared with placebo and aclidinium alone (all *p* < 0.05). Reductions in early-morning symptom severity were also observed for aclidinium/formoterol versus formoterol in Baseline Dyspnea Index-defined more symptomatic patients (*p* < 0.01)  For less symptomatic patients, reductions in early-morning symptom severity were demonstrated for aclidinium/formoterol versus aclidinium (*p* < 0.05)  In the total pooled population, an improvement in limitations of early-morning activity was observed with aclidinium/formoterol versus placebo and monotherapies (*p* < 0.05) |
| Partridge^45^; 2009 | 442 | 1. Budesonide/formoterol 320/9 µg BID 2. Salmeterol/fluticasone 50/500 µg BID | Global Chest Symptoms Questionnaire (GCSQ)  Capacity of Daily Living during the Morning questionnaire (CDLM)  Clinical COPD questionnaire (CCQ) | Improvements in symptom scores within 15 minutes after drug administration were similar for both drugs, but budesonide/formoterol treatment resulted in significantly greater improvements in total morning activities score (getting washed, dried, dressed, eating breakfast and walking around the home; *p* < 0.05)  On comparing patients’ abilities to perform morning activities, treatment with budesonide/formoterol resulted in statistically significant improvements compared with salmeterol/fluticasone in total score on the CDLM questionnaire (*p* < 0.05)  Overall CCQ scores were comparable between the budesonide/formoterol and salmeterol/fluticasone treatment arms, with no significant differences |
| Resiner^46^; 2017 | 123 | 1. Glycopyrrolate/formoterol fumarate 18/9.6 µg BID 2. Tiotropium 5 µg 3. Placebo | Spirometry | Glycopyrrolate/formoterol fumarate (GFF) metered-dose inhaler (MDI) significantly improved 24-h lung function versus placebo, with similar benefits in the second 12-h period compared with the first, supporting twice-daily dosing of GFF MDI |
| Singh^47^; 2014 | 1,729 | 1. Aclidinium/formoterol 400/12 µg BID 2. Aclidinium/formoterol 400/6 µg BID 3. Aclidinium 400 µg BID 4. Formoterol 12 µg BID 5. Placebo | COPD symptoms questionnaire (via eDiary)  E-RS questionnaire | The improvements in overall E-RS symptoms with both aclidinium/formoterol doses were significantly greater compared with the monotherapies and placebo (all comparisons, *p* < 0.05)  Changes from baseline in early-morning symptoms scores followed a similar pattern, although not all comparisons reached statistical significance |
| Ulrik^48^; 1995 | 63 | 1. Salmeterol 50 µg BID 2. Placebo | Patient daily diary cards | Median daytime symptom scores were significantly lower during the salmeterol period than during the placebo period (*p* < 0.001 for both), whereas no difference in median percentage of days or nights with a symptom score of 0 was found between the 2 treatment periods |
| van Noord^49^; 2000 | 144 | 1. Salmeterol 50 µg BID 2. Salmeterol 50 µg BID/ipratropium 40 µg QID 3. Placebo | Patient daily diary cards | Throughout the treatment period the mean decrease in daytime symptoms was non-significant with placebo, but statistically significant with both active treatment groups (*p* < 0.001)  In terms of the percentage of days with a minimal level of symptoms, improvements in both active treatment groups were better than in the placebo group (*p* < 0.05), but no significant difference was observed between the salmeterol and the salmeterol/ipratropium groups |
| Vincken^50^; 2014 | 449 | 1. Indacaterol 150 µg + glycopyrronium 50 µg 2. Indacaterol 150 µg and placebo | Patient daily diary cards (via eDiary) | Over the 12-week treatment period, diary-card data showed a significant improvement in the percentage of days able to perform usual activities in patients receiving indacaterol and glycopyrronium, compared with patients receiving indacaterol and placebo (*p =*0.016)  Significant differences were also observed in change from baseline in mean daytime respiratory symptom score (*p =*0.025), but there was no difference between the treatments in terms of change from baseline in mean daily total symptom scores |
| Vogelmeier^51^; 2013 | 523 | 1. Indacaterol/glycopyrronium 110/50 µg (QVA149) 2. Fluticasone propionate/salmeterol 250/50 µg BID (FSC) | eDiary | Reductions in patient symptom scores were not significantly different between treatment groups  The percentage of days with no daytime symptoms over 26 weeks was significantly better for QVA149 (*p =*0.049)  Mean daytime breathlessness scores showed significant reductions for QVA149 versus FSC (*p =*0.021) |
| Welte^52^; 2009 | 660 | 1. Budesonide/formoterol 320/9 µg BID + tiotropium 18 µg 2. Placebo + tiotropium 18 µg | eDiary  GCSQ  CDLM | Treatment differences were demonstrated in all COPD symptom scores (breathlessness, night-time awakening, chest tightness, and cough) in favor of budesonide/formoterol added to tiotropium (all *p* < 0.001)  Using the GCSQ, significant benefits of budesonide/formoterol + tiotropium over placebo + tiotropium were seen in terms of 5 and 15 minutes post-dose breathlessness and chest tightness (*p* < 0.05)  Significant improvements in morning activities (CDLM total score and for all individual questions except “time to finish morning activities”) were also seen with budesonide/formoterol added to tiotropium versus tiotropium alone at Week 12 (*p* < 0.05) |

^#^: comprises 11 respiratory items from the 14-item EXAcerbations of Chronic pulmonary disease Tool [EXACT] questionnaire; ^¶^: measures COPD symptoms during the early morning and at night (+ rescue medication use); ^+^: represents morning assessment of the COPD eDiary (previously validated in patients with COPD).

BID, twice daily; QID, four times daily; QD, once daily.

**References**

1. Ali Zohal, M., Yazdi, Z. & Kazemifar, A. M. Daytime sleepiness and quality of sleep in patients with COPD compared to control group. *Glob J Health Sci* **5**, 150-155 (2013).

2. Bateman, E. D. *et al*. Aclidinium bromide and formoterol fumarate as a fixed-dose combination in COPD: pooled analysis of symptoms and exacerbations from two six-month, multicentre, randomised studies (ACLIFORM and AUGMENT). *Respir Res* **16**, 92 (2015).

3. Globe, G. *et al*. Development of the chronic obstructive pulmonary disease morning symptom diary (COPD-MSD). *Health Qual Life Outcomes* **14**, 104 (2016).

4. Kim, Y. J. *et al*. Patient's perception of symptoms related to morning activity in chronic obstructive pulmonary disease: the SYMBOL study. *Korean J Intern Med* **27**, 426-435 (2012).

5. Kulich, K., Keininger, D. L., Tiplady, B. & Banerji, D. Symptoms and impact of COPD assessed by an electronic diary in patients with moderate-to-severe COPD: psychometric results from the SHINE study. *Int J Chron Obstruct Pulmon Dis* **10**, 79-94 (2015).

6. Kuyucu, T. *et al*. A cross-sectional observational study to investigate daily symptom variability, effects of symptom on morning activities and therapeutic expectations of patients and physicians in COPD-SUNRISE study. *Tuberk Toraks* **59**, 328-339 (2011).

7. Lu, M. *et al*. Perception of circadian variation of symptoms in Chinese patients with chronic obstructive pulmonary disease. *J Thorac Dis* **9**, 3888-3895 (2017).

8. Marth, K., Schuller, E. & Pohl, W. Improvements in patient-reported outcomes: a prospective, non-interventional study with aclidinium bromide for treatment of COPD. *Respir Med* **109**, 616-624 (2015).

9. Miravitlles, M. *et al*. The relationship between 24-hour symptoms and COPD exacerbations and healthcare resource use: results from an observational study (ASSESS). *COPD* **13**, 561-568 (2016).

10. Miravitlles, M. *et al*. Observational study to characterise 24-hour COPD symptoms and their relationship with patient-reported outcomes: results from the ASSESS study. *Respir Res* **15**, 122 (2014).

11. Miravitlles, M. *et al*. The variability of respiratory symptoms and associated factors in COPD. *Respir Med* **129**, 165-172 (2017).

12. Munoz, A., Small, M., Wood, R., Ribera, A. & Nuevo, J. The impacts of morning, daytime, and nighttime symptoms on disease burden in real-world patients with COPD. *Int J Chron Obstruct Pulmon Dis* **13**, 1557-1568 (2018).

13. Partridge, M. R., Karlsson, N. & Small, I. R. Patient insight into the impact of chronic obstructive pulmonary disease in the morning: an internet survey. *Curr Med Res Opin* **25**, 2043-2048 (2009).

14. Roche, N., Small, M., Broomfield, S., Higgins, V. & Pollard, R. Real world COPD: association of morning symptoms with clinical and patient reported outcomes. *COPD* **10**, 679-686 (2013).

15. Soler-Cataluna, J. J. *et al*. Prevalence and perception of 24-hour symptom patterns in patients with stable chronic obstructive pulmonary disease in Spain. *Arch Bronconeumol* **52**, 308-315 (2016).

16. Stephenson, J. J. *et al*. Impact and factors associated with nighttime and early morning symptoms among patients with chronic obstructive pulmonary disease. *Int J Chron Obstruct Pulmon Dis* **10**, 577-586 (2015).

17. Tsiligianni, I., Metting, E., van der Molen, T., Chavannes, N. & Kocks, J. Morning and night symptoms in primary care COPD patients: a cross-sectional and longitudinal study. An UNLOCK study from the IPCRG. *NPJ Prim Care Respir Med* **26**, 16040 (2016).

18. van Buul, A. R., Kasteleyn, M. J., Chavannes, N. H. & Taube, C. The association between objectively measured physical activity and morning symptoms in COPD. *Int J Chron Obstruct Pulmon Dis* **12**, 2831-2840 (2017).

19. Decramer, M. *et al*. COPD awareness survey: do Belgian pulmonary physicians comply with the GOLD guidelines 2010? *Acta Clin Belg* **68**, 325-340 (2013).

20. Espinosa de los Monteros, M. J., Pena, C., Soto Hurtado, E. J., Jareno, J. & Miravitlles, M. Variability of respiratory symptoms in severe COPD. *Arch Bronconeumol* **48**, 3-7 (2012).

21. Kessler, R. *et al*. Symptom variability in patients with severe COPD: a pan-European cross-sectional study. *Eur Respir J* **37**, 264-272 (2011).

22. O'Hagan, P. & Chavannes, N. H. The impact of morning symptoms on daily activities in chronic obstructive pulmonary disease. *Curr Med Res Opin* **30**, 301-314 (2013).

23. Worth, H. *et al*. The 'real-life' COPD patient in Germany: the DACCORD study. *Respir Med* **111**, 64-71 (2016).

24. Svedsater, H. *et al*. Evaluation and quantification of treatment preferences for patients with asthma or COPD using discrete choice experiment surveys. *Respir Med* **132**, 76-83 (2017).

25. Tabak, M. *et al*. Telemonitoring of daily activity and symptom behavior in patients with COPD. *Int J Telemed Appl* **2012**, 438736 (2012).

26. van Buul, A. R., Kasteleyn, M. J., Chavannes, N. H. & Taube, C. Physical activity in the morning and afternoon is lower in patients with chronic obstructive pulmonary disease with morning symptoms. *Respir Res* **19**, 49 (2018).

27. Beier, J. *et al*. Efficacy and safety of aclidinium bromide compared with placebo and tiotropium in patients with moderate-to-severe chronic obstructive pulmonary disease: results from a 6-week, randomized, controlled Phase IIIb study. *COPD* **10**, 511-522 (2013).

28. Beier, J., Mroz, R., Kirsten, A. M., Chuecos, F. & Gil, E. G. Improvement in 24-hour bronchodilation and symptom control with aclidinium bromide versus tiotropium and placebo in symptomatic patients with COPD: post hoc analysis of a Phase IIIb study. *Int J Chron Obstruct Pulmon Dis* **12**, 1731-1740 (2017).

29. Bleecker, E. R., Emmett, A., Crater, G., Knobil, K. & Kalberg, C. Lung function and symptom improvement with fluticasone propionate/salmeterol and ipratropium bromide/albuterol in COPD: response by beta-agonist reversibility. *Pulm Pharmacol Ther* **21**, 682-688 (2008).

30. Dahl, R. *et al*. Safety and efficacy of dual bronchodilation with QVA149 in COPD patients: the ENLIGHTEN study. *Respir Med* **107**, 1558-1567 (2013).

31. Donohue, J. F., Kalberg, C., Emmett, A., Merchant, K. & Knobil, K. A short-term comparison of fluticasone propionate/salmeterol with ipratropium bromide/albuterol for the treatment of COPD. *Treat Respir Med* **3**, 173-181 (2004).

32. Donohue, J. F. *et al*. Once-daily bronchodilators for chronic obstructive pulmonary disease: indacaterol versus tiotropium. *Am J Respir Crit Care Med* **182**, 155-162 (2010).

33. D'Urzo, A. D. *et al*. In patients with COPD, treatment with a combination of formoterol and ipratropium is more effective than a combination of salbutamol and ipratropium: a 3-week, randomized, double-blind, within-patient, multicenter study. *Chest* **119**, 1347-1356 (2001).

34. D'Urzo, A. *et al*. Efficacy and safety of fixed-dose combinations of aclidinium bromide/formoterol fumarate: the 24-week, randomized, placebo-controlled AUGMENT COPD study. *Respir Res* **15**, 123 (2014).

35. D'Urzo, A. *et al*. Once daily glycopyrronium for the treatment of COPD: pooled analysis of the GLOW1 and GLOW2 studies. *Curr Med Res Opin* **30**, 493-508 (2014).

36. Jones, P. W. *et al*. The effect of aclidinium bromide on daily respiratory symptoms of COPD, measured using the Evaluating Respiratory Symptoms in COPD (E-RS: COPD) diary: pooled analysis of two 6-month Phase III studies. *Respir Res* **17**, 61 (2016).

37. Kerwin, E. M. *et al*. Efficacy and safety of a 12-week treatment with twice-daily aclidinium bromide in COPD patients (ACCORD COPD I). *COPD* **9**, 90-101 (2012).

38. Kostikas, K. *et al*. Early changes in eDiary COPD symptoms predict clinically relevant treatment response at 12 weeks: analysis from the CRYSTAL study. *COPD* **15**, 185-191 (2018).

39. LaForce, C. *et al*. Efficacy and safety of twice-daily glycopyrrolate in patients with stable, symptomatic COPD with moderate-to-severe airflow limitation: the GEM1 study. *Int J Chron Obstruct Pulmon Dis* **11**, 1233-1243 (2016).

40. Magnussen, H. *et al*. Indacaterol once-daily is equally effective dosed in the evening or morning in COPD. *Respir Med* **104**, 1869-1876 (2010).

41. Make, B. *et al*. The efficacy and safety of inhaled fluticasone propionate/salmeterol and ipratropium/albuterol for the treatment of chronic obstructive pulmonary disease: an eight-week, multicenter, randomized, double-blind, double-dummy, parallel-group study. *Clin Ther* **27**, 531-542 (2005).

42. Marin, J. M. *et al*. Early bronchodilator action of glycopyrronium versus tiotropium in moderate-to-severe COPD patients: a cross-over blinded randomized study (Symptoms and Pulmonary function in the moRnING). *Int J Chron Obstruct Pulmon Dis* **11**, 1425-1434 (2016).

43. McGarvey, L. *et al*. Effect of aclidinium bromide on cough and sputum symptoms in moderate-to-severe COPD in three phase III trials. *BMJ Open Respir Res* **3**, e000148 (2016).

44. Miravitlles, M., Chapman, K. R., Chuecos, F., Ribera, A. & Gil, E. G. The efficacy of aclidinium/formoterol on lung function and symptoms in patients with COPD categorized by symptom status: a pooled analysis. *Int J Chron Obstruct Pulmon Dis* **11**, 2041-2053 (2016).

45. Partridge, M. R., Schuermann, W., Beckman, O., Persson, T. & Polanowski, T. Effect on lung function and morning activities of budesonide/formoterol versus salmeterol/fluticasone in patients with COPD. *Ther Adv Respir Dis* **3**, 1-11 (2009).

46. Reisner, C. *et al*. 24-h bronchodilation and inspiratory capacity improvements with glycopyrrolate/formoterol fumarate via co-suspension delivery technology in COPD. *Respir Res* **18**, 157 (2017).

47. Singh, D. *et al*. Efficacy and safety of aclidinium bromide/formoterol fumarate fixed-dose combinations compared with individual components and placebo in patients with COPD (ACLIFORM-COPD): a multicentre, randomised study. *BMC Pulm Med* **14**, 178 (2014).

48. Ulrik, C. S. Efficacy of inhaled salmeterol in the management of smokers with chronic obstructive pulmonary disease: a single centre randomised, double blind, placebo controlled, crossover study. *Thorax* **50**, 750-754 (1995).

49. van Noord, J. A. *et al*. Long-term treatment of chronic obstructive pulmonary disease with salmeterol and the additive effect of ipratropium. *Eur Respir J* **15**, 878-885 (2000).

50. Vincken, W. *et al*. Efficacy and safety of coadministration of once-daily indacaterol and glycopyrronium versus indacaterol alone in COPD patients: the GLOW6 study. *Int J Chron Obstruct Pulmon Dis* **9**, 215-228 (2014).

51. Vogelmeier, C. F. *et al*. Efficacy and safety of once-daily QVA149 compared with twice-daily salmeterol-fluticasone in patients with chronic obstructive pulmonary disease (ILLUMINATE): a randomised, double-blind, parallel group study. *Lancet Respir Med* **1**, 51-60 (2013).

52. Welte, T. *et al*. Efficacy and tolerability of budesonide/formoterol added to tiotropium in patients with chronic obstructive pulmonary disease. *Am J Respir Crit Care Med* **180**, 741-750 (2009).
